# Prognostic value of Dickkopf-1 and ß-catenin expression according to the antitumor immunity of CD8-positive tumor-infiltrating lymphocytes in biliary tract cancer

**DOI:** 10.1038/s41598-022-05914-4

**Published:** 2022-02-04

**Authors:** Seo Ree Kim, Hye Sung Won, Ji Hyun Yang, Der Sheng Sun, Kwangil Yim, Mineui Hong, Soon Auck Hong, Jung-Sook Yoon, Sang Hoon Chun, Kee-Hwan Kim, Yoon Ho Ko

**Affiliations:** 1 Division of Oncology, Department of Internal Medicine, College of Medicine, The Catholic University of Korea, Seoul, Republic of Korea; 2 Cancer Research Institute, College of Medicine, The Catholic University of Korea, Seoul, Republic of Korea; 3 Department of Hospital Pathology, Uijeongbu St. Mary’s Hospital, College of Medicine, The Catholic University of Korea, Seoul, Republic of Korea; 4 Department of Pathology, College of Medicine, Chung-Ang University, Seoul, Republic of Korea; 5 Uijeongbu St. Mary’s Hospital Clinical Research Laboratory, The Catholic University of Korea, Seoul, Republic of Korea; 6 Department of Surgery, Uijeongbu St. Mary’s Hospital, College of Medicine, The Catholic University of Korea, Seoul, Republic of Korea

**Keywords:** Tumour immunology, Biliary tract cancer

## Abstract

The role of β-catenin and Dickkopf-1 (DKK1) is dependent on the specific immunobiology of T cell inflammation in biliary tract cancer (BTC). We aimed to analyze the role of DKK1 or β-catenin as a prognostic factor in BTC, and determine the clinical associations of ß-catenin and DKK1 with CD8+ tumor-infiltrating lymphocytes (TIL). We used data from The Cancer Genome Atlas Research Network and the clinicopathological data of 145 patients with BTC who had undergone primary radical resection between 2006 and 2016. CD8+ TIL expression was a significant predictor of favorable overall survival (OS) and relapse-free survival (RFS) (median OS, 34.9 months in high-TIL, 16.7 months in low-TIL, *P* < 0.0001 respectively; median RFS, 27.1 months in high-TIL, 10.0 months in low-TIL, *P* < 0.0001 respectively). In the high-CD8+ TIL BTC group, the tumor expression of β-catenin and DKK1 had a significant negative impact on either OS or RFS. In the low-TIL BTC group, there were no differences according to ß-catenin and DKK1 expression. Cox regression multivariate analysis demonstrated that CD8+ TIL and β-catenin retained significant association with OS. Among patients with resected BTC, the β-catenin and DKK1 protein and high CD8+ TIL levels were associated with poor and good clinical outcomes, respectively.

## Introduction

Biliary tract cancer (BTC) accounts for approximately 3% of all gastrointestinal malignancies and is the most common hepatobiliary cancer after hepatocellular carcinoma^1^. BTC is a fatal and rare adenocarcinoma that arises in the biliary tree, including the intrahepatic (ICC) and extrahepatic (ECC) bile duct and gallbladder (GBC)^2,3^. Surgical resection is the primary treatment modality for early-stage BTC, but the majority of patients still develop a recurrence^4^. Further, the mortality rate is high primarily because patients are diagnosed at a late stage^5^. Despite recent advancements in our understanding of the molecular biology of BTC, limited progress has been made in cytotoxic systemic therapy for this disease. Agents targeting IDH1, fibroblast growth factor receptor, and EGFR have demonstrated only limited efficacy^5–7^. In addition, immunotherapy has minimal usefulness in BTC, showing only modest benefits in advanced BTC patients with deficient mismatch repair and high microsatellite instability status^8^. Therefore, new therapeutic molecular studies are required to improve the survival of patients by overcoming the heterogeneity of BTC.

The Wnt/β-catenin signaling cascade controls cell proliferation, cell polarity, and cell fate during embryonic development and homeostasis in human tissues^9^. The mutational status of this pathway’s components is highly relevant to the pathogenesis of BTC^2^. Particularly, activation of the Wnt pathway has been shown to be associated with chemoresistance and metastatic spread in ECC and ICC^10,11^. Different mutations found in ECC and ICC sequencing are known to act on Wnt/β-catenin to induce chromosomal instability and oncogenesis^12,13^. Some genetic variants in the Wnt/β-catenin pathway are associated with decreased apoptosis of GBC and influenced susceptibilities^2^.

Dickkopf-related protein1 (DKK1), the most well-known Wnt antagonist of the canonical Wnt/β-catenin pathway, is a 35-kDa protein that contains a secreted signal peptide sequence. DKK1 can inhibit Wnt signaling by interacting with the Wnt receptors of the Frizzled family^14^. DKK1 plays a role in cancer proliferation, invasion, and growth via the modulation of Wnt signaling^14,15^. DKK1 is expressed in multiple tumor types, including BTC, but its impact and the clinical role of this pathway’s components are not completely understood^9,16^. Recent studies have suggested that high DKK1 expression in BTC is associated with immunosuppressive conditioning through myeloid-derived suppressor cells(MDSC) and tumor-associated macrophages^17^. It is also associated with high expression of matrix metalloproteinase 9 proteins, which play a role in tumor cell invasion, angiogenesis, and lymph node metastasis in BTC and are correlated with poor prognosis^15,16,18^.

Given that BTC is constantly exposed to intestinal microbial products, it has both the immune tolerance capability to suppress inappropriate inflammatory responses and the immune capability to protect against harmful stimuli, such as infection and tumor^19–21^. Several studies have demonstrated that BTC contains abundant tumor-infiltrating lymphocytes (TILs) and cancer-associated fibroblasts in the tumor microenvironment (TME)^19,21^. In addition, Wnt/β-catenin signaling and DKK1 expression are associated with immunosuppressive modulation and protumoral conditioning in BTC through several mechanisms^19^, including activation of innate immune cells such as tumor-associated macrophages or MDSCs^21–23^ or loss of effector functions by T cell de-differentiation^24^. However, the clinical implications for β-catenin according to tumor infiltrative immune cells and DKK1, as an antagonist of Wnt/β-catenin signaling, remains controversial. Therefore, this study aimed to investigate the role of DKK1 and β-catenin as prognostic factors in BTC with reference to CD8+ TILs.

## Materials and methods

### Wnt/β-catenin gene expression according to CD8 in The Cancer Genome Atlas BTC database manuscript formatting

This retrospective study analyzed data from The Cancer Genome Atlas (TCGA) Research Network to identify the associations of Wnt/β-catenin gene expression in CD8 cells and clinical outcomes in BTC. mRNA expression data and clinical data were downloaded from the TCGA data portal (https://tcga-data.nci.nih.gov/tcga/). All mRNA expression data were log2-transformed. The expressions of CD8A, DKK1, and CTNNB1 genes were analyzed in 45 samples.

Study population. To validate the findings from TCGA BTC datasets, we analyzed the clinicopathological data of 145 patients with BTC who had undergone primary radical resection between 2006 and 2016 at Uijeongbu St. Mary’s Hospital of the Catholic University of Korea. BTC was classified into ICC; ECC; and GBC, including perihilar cholangiocarcinoma and distal common bile duct cancer. The inclusion criteria were (1) presence of pathologically confirmed biliary tract adenocarcinoma, (2) treatment by radical resection without preoperative radiation or chemotherapy, and (3) availability of paraffin-embedded tumor specimens. Postoperative pathological staging was based on the American Joint Committee on Cancer staging criteria, 8th edition. Patient data, including age, sex, date of diagnosis, recurrence, and death, were retrieved from the electronic medical records.

### Immunohistochemistry

Immunohistochemistry was performed on formalin-fixed, paraffin-embedded tissue sections. Whole tissue sections of representative tumor samples were used for antigen retrieval and incubated with human-specific antibodies against DKK1 (1: 200, Abcam, Cambridge, UK) and β-catenin (1:100, Cell Signaling, Danvers, MA, USA). The intensity of DKK1 staining was scored as follows: 0, no staining; 1, weakly positive; 2, moderately positive; and 3, strongly positive. The proportion of DKK1-stained cells was calculated as the percentage of positive tumor cells. H scores, ranging from 0 to 300, were determined by multiplying the intensity score by the proportion of DKK1-stained cells. For the characterization of the inflammatory components, sections were incubated with primary antibodies: CD8 (1:100, C8/144B, Dako, Cambridge, UK) following the manufacturer’s instructions. ß-catenin protein immunoreaction was considered positive if more than 10% of tumor cells showed nuclear and cytoplasmic staining^9^. The cut-off H score for high DKK1 expression was set at 80 using maximally selected rank statistics (quartile) calculated using R statistical programming, version 3.2.3 (https://www.r-project.org/), and high CD8 positivity was set at 32 (median), following previous reference articles^9,25^.

### Statistical analysis

Categorical variables were compared using the chi-square test and Fisher’s exact test, as needed. Overall survival (OS) was defined as the time from diagnosis to any-cause death or the last follow-up. Relapse-free survival (RFS) was calculated from the date of diagnosis to the date of the first distant or local disease recurrence or the last follow-up. Survival curves were generated using the Kaplan–Meier method and compared using the log-rank test by GraphPad Prism 8.0 (GraphPad Software, Inc., San Diego, CA, USA). Cox proportional hazards regression models were used to identify the significance of prognostic factors. Survival rates and hazard ratios (HRs) were shown with their respective 95% confidence intervals (CIs). All statistical analyses were performed using R statistical programming (version 3.4.1) and the SPSS software package (version 23, SPSS, Chicago, IL, USA). A two-sided P-value of < 0.05 was considered statistically significant in all tests and models.

### Ethics approval and consent to participate

This study was approved by the Institutional Research Ethics Board of Uijeongbu St. Mary’s Hospital of the Catholic University of Korea (UC15SISI0155) and adhered to the Declaration of Helsinki. Written informed consent was obtained from all subjects involved in the study. Patient anonymity was preserved.

## Results

### Gene expression in TCGA

Neither DKK1 nor CTNNB1 expression was associated with CD8A gene expression (Supplementary Fig. S1). There was no significant relationship between DKK1 and CTNNB1 gene expression, even in the analysis stratified by CD8A expression (Supplementary Fig. S2). However, high CD8A and CTNNB1 gene expressions were correlated with clinical outcomes, as expected (Supplementary Fig. S3). Interestingly, the clinical role of the CTNNB1 gene was only observed in patients with high CD8A gene expression (Supplementary Fig. S4). This showed that low β-catenin expression tends to have a good prognostic role only in high CD8+ T cell conditioning. β-catenin signaling and DKK1 expression indicated mutual and exclusively poor clinical outcomes, and that immunobiology, such as CD8+ T cells, was involved in the process.

### Patient characteristics

Baseline characteristics of the 145 patients are summarized in Table 1. The median age was 67.1 years (range 31–87 years), and 46.9% of the patients were male. Among them, 64.8% (n = 94), 22.8% (n = 33), and 12.4% (n = 18) had GBC, ECC, and ICC, respectively. Pathologically, 52 (35.9%) patients had stage T1 or T2 disease; 28 (19.3%) patients, ≥ N1 and 72 (49.7%) patients, pathological stage III or IV. The median follow-up period after curative surgical resection was 32.1 months (range 2.4 to 138.3 months). Overall, 86 (59.3%) patients were dead, while 59 (40.7%) patients were alive at the last follow-up. Disease recurrence was observed in 101 patients (69.7%).

**Table 1:** The correlation of clinicopathologic findings of BTC with DKK1, beta catenin with CD8 tumor infiltrating.

variables	No. of patients (%)	CD8+ TILs		*P* value
		Low, n = 72 (%)	High, n = 73 (%)	
Age				0.776
≤ 65	52 (35.9)	25 (17.2)	27 (18.6)	
≥ 65	93 (64.1)	47 (32.4)	46 (31.7)	
Sex				0.557
Male	68 (46.9)	32 (22.1)	36 (24.8)	
Female	77 (53.1)	40 (27.6)	37 (25.5)	
Primary site				0.040
GBC	94 (64.8)	42 (29.0)	52 (35.9)	
ECC*	33 (22.8)	17 (11.7)	16 (11.0)	
ICC	18 (12.4)	13 (9.0)	5 (3.4)	
Histologic grade				0.274
Well	54 (37.2)	24 (16.6)	30 (20.7)	
Mod/poor/un	91 (62.8)	49 (33.8)	42 (29.0)	
pT stage				0.007
≤ T2	52 (35.9)	18 (12.4)	34 (23.4)	
≥ T3	93 (64.1)	54 (37.2)	39 (26.9)	
pN stage				0.968
N0	117 (80.7)	58 (40)	59 (40.7)	
≥ N1	28 (19.3)	14 (9.7)	14 (9.7)	
pTNM stage				0.002
≤ II	73 (50.3)	27 (18.6)	46 (31.7)	
≥ III	72 (49.7)	45 (31.0)	27 (18.6)	
Lymphovascular invasion				0.053
No	88 (60.7)	38 (26.2)	50 (34.5)	
Yes	57 (39.3)	34 (23.4)	23 (15.9)	

*BTC* biliary tract cancer, *DKK1* Dickkopf relatd protein1, *No.* Number. of patients, *GBC* gallbladder cancer, *ECC* extrahepatic cholangiocarcinoma, *ICC* intrahepatic cholangiocarcinoma, *pT stage* pathologic T stage, *pN stage* pathologic N stage, *LVI* Lymphovascular invasion.

*ECC includes perihilar cholangiocarcinoma, common bile duct cancer and distal common bile duct cancer.

p^¶^, < 0.05, was considered statistically significant. All variables were compared using the chi-square test and Fisher’s exact test.

### Marker expression and its relationships with clinicopathological findings

**Figure 1:**
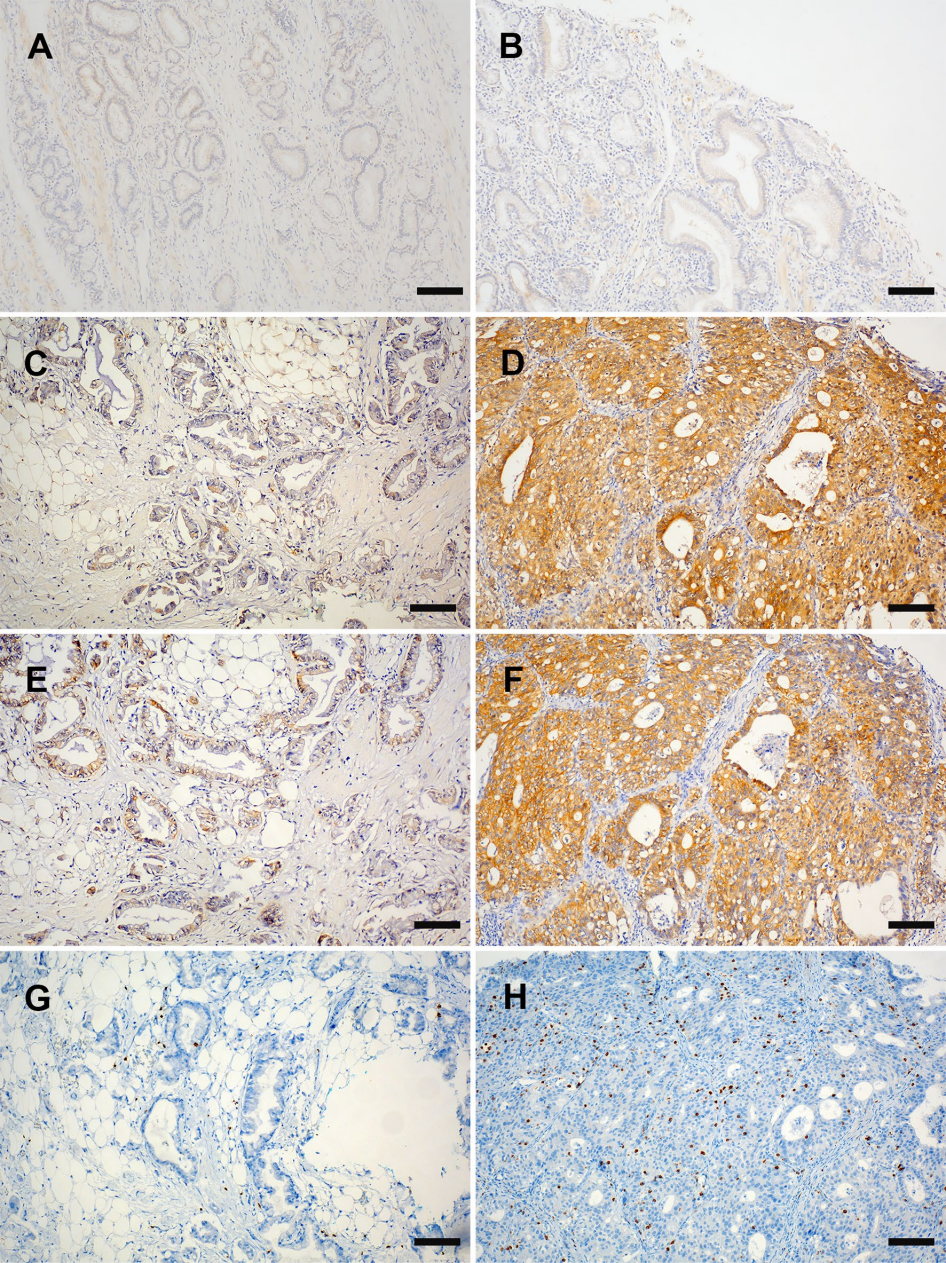
Immunohistochemical staining of DKK1, β-catenin, and CD8. Representative images (**C**, **E**, and **G**), (**D**, **F**, and **H**) are derived from the same tissue blocks. (**A**) DKK1 and (**B**) β-catenin expression in the normal bile duct mucosa, (**C**) low and (**D**) high expression of DKK1, (**E**) low and (**F**) high expression of β-catenin, (**G**) low and (**H**) high density of CD8+ tumor-infiltrating lymphocytes. (Original magnification, × 100; scale bar, 100 mm).

In the non-neoplastic biliary epithelium, only scant or weak cytoplasmic staining of DKK1 and ß-catenin was detected (Fig. 1A,B). High expression of DKK1 and β-catenin positivity were observed in 37 (25.5%) and 40 (27.6%) tumors, respectively (Fig. 1C–F). Based on the median cut-off score for CD8 expression, the patients were classified into high (n = 73) and low (n = 72) CD8+ TIL groups (Fig. 1G,H). Low CD8 expression was significantly associated only with advanced T stage (*P* = 0.007), advanced TNM stage (*P* = 0.002), and BTC other than GBC (*P* = 0.040) (Table 1).

With respect to marker expression, β-catenin or DKK1 positivity was not related to CD8 expression (Supplementary Fig. S5). High DKK1 expression was significantly associated with advanced T stage regardless of CD8 expression (low-CD8+ TIL, *P* = 0.029; high-CD8+ TIL, *P* = 0.036, respectively). However, there was no association between ß-catenin positivity and any clinicopathological factor based on CD8 expression (Table 2).

**Table 2:** Correlation between clinicopathologic findings and DKK1 & β catenin level according CD8 expression.

Clinico pathologic feature	CD8 Low expression		CD8 High expression		CD8 Low expression		CD8 High expression	
	DKK1 Low	DKK1 High	DKK1 Low	DKK1 High	β catenin off	β catenin on	β catenin off	β catenin on
pT stage								
≤ T2	16	2	31	3	13	5	26	8
≥ T3	33	21	28	11	43	11	23	16
*p* value		0.029		0.036		0.513		0.112
pN stage								
N0	39	19	49	10	46	12	39	20
≥ N1	10	4	10	4	10	4	10	4
*p* value		0.763		0.321		0.524		0.703
pTNM stage								
≤ II	21	6	40	6	21	6	32	14
≥ III	28	17	19	8	35	10	17	10
*p* value		0.171		0.082		1.000		0.562
Histologic grade								
Well	17	6	29	2	19	4	25	6
Mod/poor/un	32	17	30	12	37	12	24	18
*p* value		0.465		0.018		0.499		0.035

*DKK1* Dickkopf relatd protein1, *pT stage* pathologic T stage, *pN stage* pathologic N stage.

Histologic grade, mod/poor/un, moderate differentiated/ poorly differentiated/ undifferentiated.

**Figure 2:**
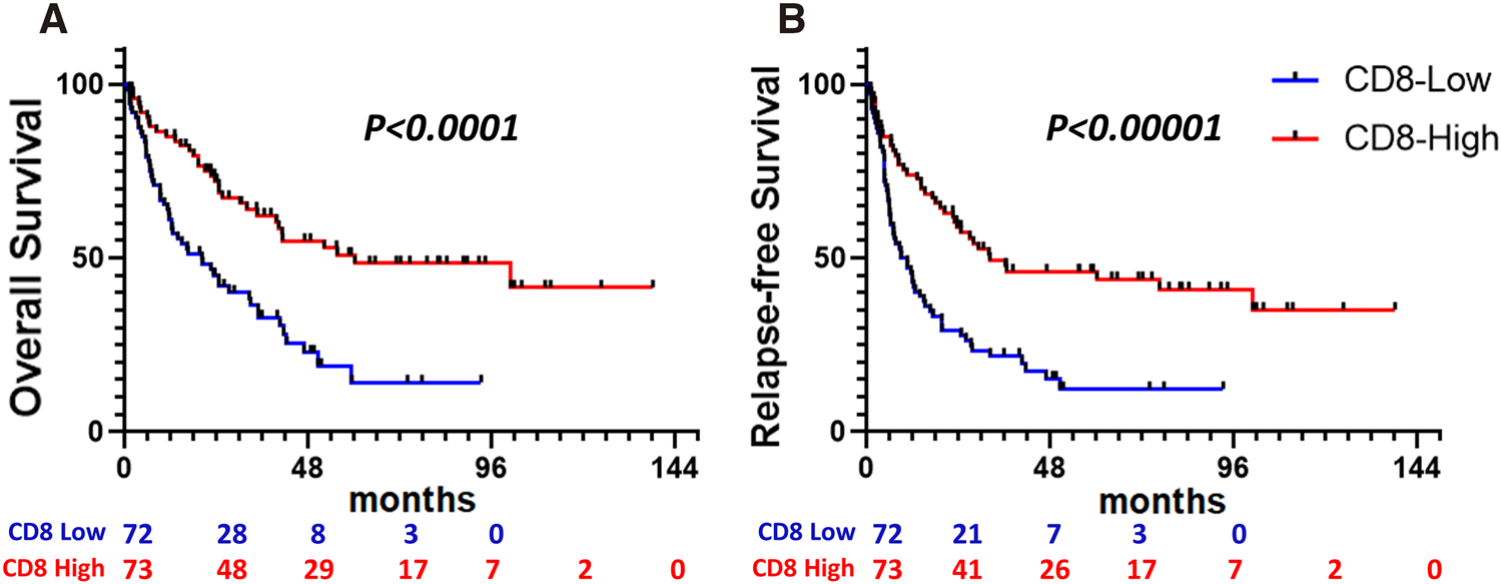
Overall survival (**A**) and relapse-free survival (**B**) according to CD8 expression.

Prognostic value of CD8, β-catenin, and DKK1 expression in bile duct cancer. The overall 5-year OS rate was 16.5% and the median OS time was 24.7 months (range 1.3–138.3 months). Meanwhile, the overall 5-year RFS rate was 14.4% and the median RFS time was 18.2 months (range, 0.9–138.3 months). Analysis of the correlation between marker expression and prognosis showed that TIL expression was a significant predictor of favorable OS and RFS, as expected (median OS, 34.9 months in high-TIL, 16.7 months in low-TIL, *P* < 0.0001, respectively; median RFS, 27.1 months in high-TIL, 10.0 months in low-TIL, *P* < 0.0001, respectively) (Fig. 2A, B). Patients with positive ß-catenin expression tended to have a shorter OS (median OS, 23.95 months in positive ß-catenin, 26.1 months in negative ß-catenin, *P* = 0.1009, respectively) than those with negative expression, but the difference was not significant (Supplementary Fig. S6A,B). Meanwhile, patients with high DKK1 expression showed significantly shorter OS (median OS, 19.4 months in high DKK1, 31.7 months in low DKK1, *P* = 0.0093 respectively), but not RFS (*P* = 0.2924) (Supplementary Fig. S4C,D).

In the univariate analyses, patients who were older (*P* = 0.009), had poorly differentiated grade (*P* < 0.001), non-GBC primary site (*P* = 0.036), positive margin (*P* = 0.015), advanced stage (*P* < 0.001), lymphatic invasion status (*P* < 0.001), and perineural invasion status (*P* < 0.001) predicted poor RFS (Table 3). Of these, age (HR 1.727, 95% CI 1.092–2.733; *P* = 0.020), positive margin (HR 1.686, 95% CI 1.083–2.623; *P* = 0.021), advanced stage (HR 1.816, 95% CI 1.106–2.982; *P* = 0.018) and perineural invasion status (HR 1.960, 95% CI 1.187–3.237; *P* = 0.009) were independent prognostic factors of poor RFS in the multivariate analysis. A shorter OS was found to be significantly associated with older age (*P* = 0.006), poorly differentiated grade (*P* < 0.001), positive margin (*P* = 0.022), advanced stage (*P* < 0.001), lymphatic invasion status (*P* = 0.001), and perineural invasion status (*P* < 0.001) in the univariate analysis. Risk factors that were significantly associated with shortening OS included older age (HR 1.956, 95% CI 1.198–3.194; *P* = 0.007) and advanced stage (HR 1.751, 95% CI 1.042–2.942; *P* = 0.035) in the multivariate analysis. High CD8+ TIL level was associated with good prognostic values in both RFS (HR 0.579, 95% CI 0.375–0.895; *P* = 0.014 in multivariate analysis) and OS (HR 0.477, 95% CI 0.296–0.769; *P* = 0.002 in multivariate analysis). Although, positive β-catenin and DKK1 expression did not reveal statistical significance in RFS, these were correlated with poor prognostic values in OS (HR 1.611, 95% CI 1.011–2.568; *P* = 0.045 in β-catenin; HR, 1.616, 95% CI 0.997–2.620; *P* = 0.051 in DKK1).

**Table 3:** Risk factors for overall survival & relapse free survival in 145 patients with biliary tract cancers by univariate and multivariate analyses.

Effect	OS				RFS			
	Univariate		Multivariate		Univariate		Multivariate	
	HR (95% CI)	*P* value*	HR (95% CI)	*P* value*	HR (95% CI)	*P* value*	HR (95% CI)	*P* value*
Gender, Female versus Male	0.956 (0.625–1.463)	0.837			0.980 (0.641–1.498)	0.925		
Age, ≥ 65 vs < 65	1.945 (1.211–3.123)	0.006	1.956 (1.198–3.194)	0.007	1.883 (1.173–3.021)	0.009	1.727 (1.092–2.733)	0.020
β-Catenin, on versus off	1.453 (0.927–2.278)	0.103	1.611 (1.011–2.568)	0.045	1.426 (0.909–2.239)	0.122	1.344 (0.862–2.095)	0.192
DKK(q25), High versus Low	1.848 (1.155–2.958)	0.010	1.616 (0.997–2.620)	0.051	1.716 (1.077–2.736)	0.023	1.311 (0.822–2.090)	0.256
CD8, High versus Low	0.400 (0.256–0.624)	< 0.001	0.477 (0.296–0.769)	0.002	0.405 (0.260–0.631)	< 0.001	0.579 (0.375–0.895)	0.014
Grade, mod-poor versus well	2.393 (1.481–3.867)	< 0.001	1.501 (0.891–2.529)	0.127	2.457 (1.520–3.972)	< 0.001	1.501 (0.942–2.392)	0.087
primary site, iCC&eCC versus GB	0.711 (0.461–1.096)	0.122	0.798 (0.492–1.294)	0.360	1.592 (1.032–2.457)	0.036	1.066 (0.692–1.642)	0.773
Margin, positive versus negative	1.714 (1.081–2.718)	0.022	1.524 (0.939–2.474)	0.088	1.772 (1.117–2.810)	0.015	1.686 (1.083–2.623)	0.021
pTNM stage, III-IV versus I-II	3.053 (1.946–4.791)	< 0.001	1.751 (1.042–2.942)	0.035	3.253 (2.074–5.100)	< 0.001	1.816 (1.106–2.982)	0.018
LVI, yes versus no	2.125 (1.378–3.277)	0.001	0.775 (0.442–1.358)	0.372	2.303 (1.494–3.550)	< 0.001	0.906 (0.542–1.517)	0.708
PNI, yes versus no	3.387 (2.136–5.371)	< 0.001	1.610 (0.941–2.753)	0.082	3.627 (2.294–5.735)	< 0.001	1.960 (1.187–3.237)	0.009

*OS* overall survival, *RFS* relapse-free survival, *HR* hazard ratio, *CI* confidence interval, *DKK* dickkopf.

Age,  ≥ 65 versus  < 65; Gender, female versus male; primary site, intrahepatic & extrahepatic cholangiocarcinoma (ICC & ECC) versus gallbladder (GB) cancer; tumor grade, moderate to poor versus well; Resection margin, positive(Dysplasia, Cancer) versus negative; pT, pathologic T stage, 0,T1 + 2, 1,T3 + 4; pN, pathologic N stage, 0.N0, 1.N1; pTNM Stage, pathologic TNM stage, 0.stage I-II, 1.stage III-IV; LVI, Lympho-vascular invasion, yes versus no; DKK1, High versus Low according to one quarter value; β-Catenin, on versus off; CD8, High versus Low according to median value.

P*, A two-sided *P* value of < 0.05, was considered statistically significant. Categorical variables were compared using the chi-square test.

**Figure 3:**
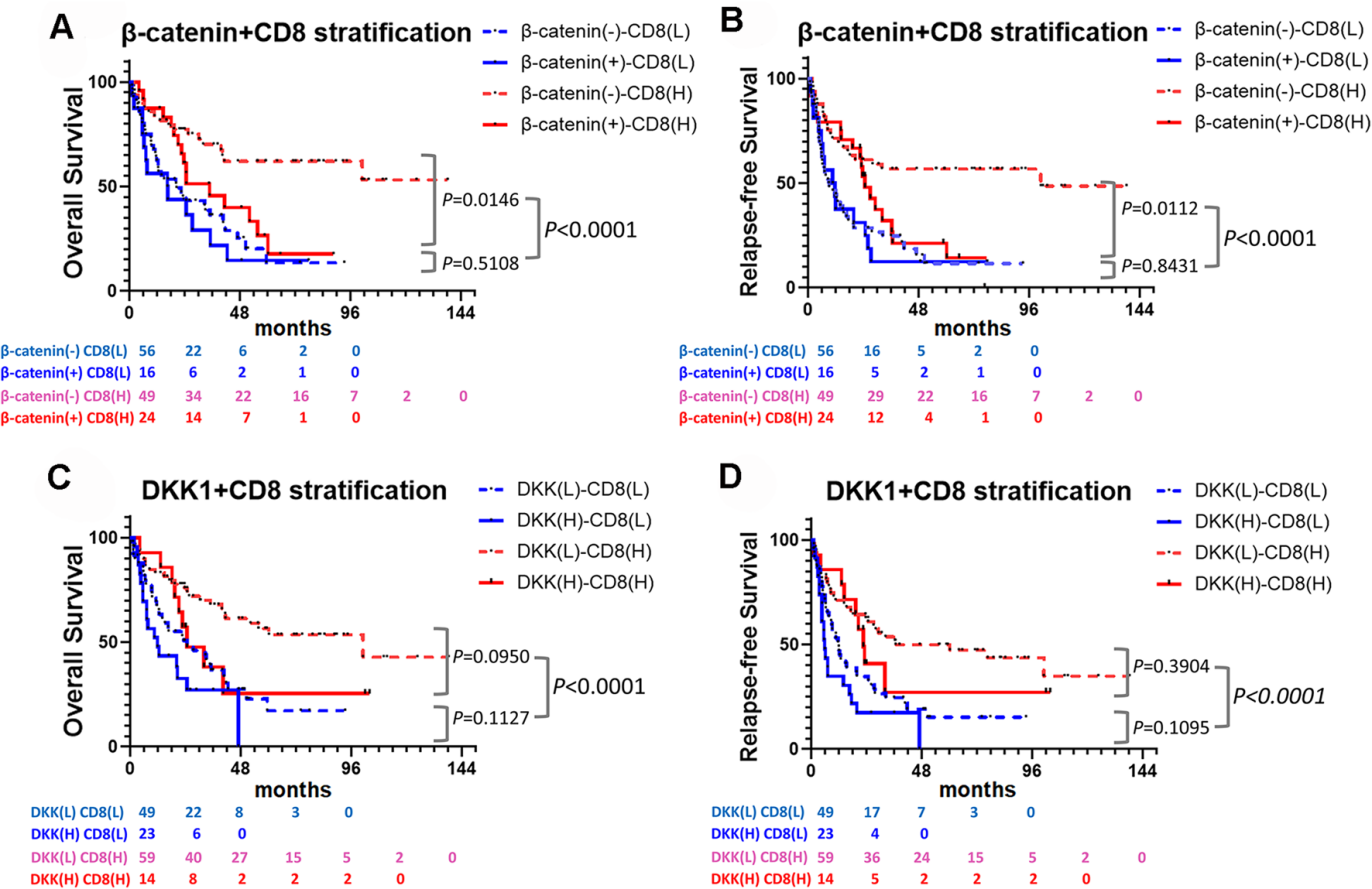
Survival curves according to ß-catenin and DKK1 expression stratified by CD8 expression (**A**, **B**). There was a significant difference in prognosis according to the presence or absence of β-catenin in the high CD8 group. Survival curves according to the difference in DKK1 level in the high and low expression CD8 groups (**C**, **D**). DKK1 was a significant predictor of prognosis in both the high and low CD8 expression groups.

The Wnt/β-catenin pathway contributes to immune evasion in tumors by suppressing the function of immune cells and attenuating CD8+ T cell infiltration^24,26^. TIL levels have prognostic values in various cancers. Therefore, to eliminate the influence of TIL expression on prognosis, we analyzed the independent prognostic values of β-catenin and DKK1 stratified by CD8+ TIL expression. Notably, the expression of β-catenin and DKK1 in tumors had a significant negative impact on OS and RFS in the CD8+ TIL-high BTC group (β-catenin: *P* = 0.0146 for OS and *P* = 0.0112 for RFS, respectively; DKK1: *P* = 0.0950 for OS and *P* = 0.3904 for RFS, respectively) (Fig. 3A,B). However, there were no differences in the OS or RFS according to β-catenin expression (OS, *P* = 0.5108 and RFS, *P* = 0.8431, respectively) and DKK1 expression (OS, *P* = 0.1127 and RFS, *P* = 0.1095, respectively) in the TIL-low BTC group (Fig. 3A–D). These findings confirmed the differential clinical role of Wnt/β-catenin proteins according to TIL expression.

The above results confirmed the difference in the extent of CD8+ T cell infiltration according to the primary tumor site, with these differences primarily being due to the immunobiological characteristics of BTC (Table 1). Therefore, we further investigated if the clinical outcomes (OS and RFS) differ according to the primary tumor site in patients with positive DKK1 and ß-catenin expression. In GBC and ECC, DKK1 and ß-catenin confirmed the good prognostic role of high CD8+ T cells, but ICC, known as immune exclusive TME, with strong immunosuppressive tendency, revealed the different roles of each marker (Supplementary Fig. S7A–F).

## Discussion

The impact of the Wnt/β-catenin pathway on cancer progression is well known. This pathway mediates immune exclusion in cancer tissue, including BTC. However, BTC has a distinct immunobiology, characterized by the presence of abundant tumor-infiltrating immune cells, owing to the constant exposure to intestinal microbes^19,20^. Furthermore, the relationship of β-catenin and DKK1 with the clinical outcomes of BTC remains unclear. In this study, a combined analysis of β-catenin and DKK1 protein expression, and CD8+ TILs showed that the expression of the β-catenin and DKK1 proteins was an independent adverse prognostic factor for BTC. In contrast, a high CD8+ TIL count was a favorable prognostic factor. Notably, the favorable clinical effect of high CD8+ TIL was observed only in BTC patients with low β-catenin or DKK1 protein expression^19,27^. These findings suggest that CD8+ T cells have a more profound prognostic impact in tumors with low Wnt/β-catenin activation.

In general, cytotoxic CD8+ T lymphocytes can recognize tumors and induce tumor cell death via the release of cytotoxic granules^28^. Thus, the degree of TIL infiltration is considered to reflect the growth, progression, and metastasis of cancer. Moreover, it is predictive of the response to cytotoxic treatments, such as chemotherapy and radiotherapy. Previous studies have demonstrated the importance of CD8+ T cell specificity and cytotoxicity^27,28^. CD8+ T cells are the most functionally representative and most abundant TILs to be found within the tumor^27–29^ High CD8+ TIL infiltration was recently shown to be correlated with better OS in patients with BTC^29,30^. Similarly, this study found that TIL expression was a strong favorable prognostic factor for OS and RFS in BTC.

The activation of the Wnt/β-catenin pathway is a hallmark of disease progression, which is associated with poor clinical outcomes in various malignancies^2,31^. In the current study, ß-catenin expression was also associated with a shorter duration of survival. The COSMIC database shows that Wnt/β-catenin activation occurs in 4.2–7.5% of cholangiocarcinoma tissue samples^32^ and in up to 57.1% of GBC tissue samples^33^. Exome sequencing analysis of 209 BTC samples from Asia and Europe showed that mutations at the CTNNB1 and APC loci are rare, unlike other gastrointestinal cancers^34^. This suggests that aberrant activation of the Wnt signaling pathway is mainly modulated by Wnt ligands and negative regulators in BTC. For example, the loss of activation of RNF43, an inhibitor of Wnt signaling, could increase Wnt signaling in the absence of mutations at the APC or CTNNB1 locus^12^. Loss of PEG3, an imprinted gene that regulates apoptosis, activates Wnt signaling, eventually leading to chromosomal instability in BTC^13^. Additionally, inflammatory macrophages are required to establish this Wnt-high state via the production of Wnt ligands^35^.

DKK1 is known to inhibit Wnt signaling by interacting with the Wnt receptors of the Frizzled family^14,36^. Moreover, DKK1 is associated with the so-called immune-desert microenvironment that results from the activation of MDSC and downregulation of natural killer cells in the cancer milieu^17,18^. However, the role of DKK1 as a tumor suppressor or oncogene varies across various malignant tumors. DKK1 overexpression has been correlated with adverse prognosis in patients with gastric, lung, and breast cancers^9,16^. We found that high DKK1 expression is associated with a poor prognosis, in accordance with these findings. Given that the Wnt signaling pathway does not serve as a binary on/off switch during tumorigenesis, it is difficult to define the role of DKK1, the modulator of the Wnt/β-catenin pathway^36^. In addition to the disruption in the negative feedback loop between DKK1 and the Wnt/β-catenin pathway^18,36^, DKK1 promotes malignancy via non-canonical Wnt pathway mechanisms^37^. DKK1 also plays a role in creating an immunosuppressive TME by activating MDSC, which downregulate the T-cell response^17,38^, as demonstrated by several in vitro studies. D'Amico et al. observed that downregulated DKK1 affects the MDSC count by rescuing β-catenin in these cells, and restores T cell recruitment at the tumor site^17^. Other studies found that DKK1 is associated with the so-called immune-desert microenvironment via the activation of MDSCs, downregulation of natural killer cells^18^ and contribution to the recruitment of Foxp3+ Treg cells, in order to restore cancer-immunological homeostasis^39^. Therefore, our clinical data on the differential roles of β-catenin and DKK1 depending on the TIL status were congruent with these experimental data.

The complex prognostic role of DKK1 remains unclear. Data from recent studies have demonstrated that DKK1 acts not only as a tumor suppressor protein, but also as an oncogene^9,18^. The influence of DKK1 on the clinical outcomes differs according to the location and TME components^40^, which is also interpreted as a dynamic effect depending on the TIL background in connection with the action of β-catenin^24^. The regulation of the Wnt/β-catenin signaling pathway is a complex process with a highly regulated hierarchy, which is influenced by both auto-regulatory and para-regulatory signals. As noted above, BTC is considered to be a heterogeneous cancer^2,30,42^. The role of Wnt/β-catenin signaling and its antagonist, DKK1, is dynamic and can vary according to the type of tumor (i.e., high-density TIL-infiltrated immune-inflamed “hot tumors” and immune-exclusive “cold tumors” with low TIL density)^19,22,23^. Wnt/β-catenin signaling is essential for T cell differentiation, effector functions, proliferation, and migration via the T-cell factor (TCF), which is the effector transcription factor of the Wnt signaling pathway^41^. As such, “hot tumors" present with more profound Wnt/β-catenin signaling and TCF modulation. Meanwhile, Wnt/β-catenin signaling and TCF modulation do not have a significant influence on the prognosis of “cold tumors” because of the insufficient substrates available for alteration in these tumors^24,26^. The differences in the resulting clinical changes in the protumoral and immunosuppressive status, according to β-catenin activation, were more prominent in the high TIL inflamed subgroup, whose clinical prognosis was comparable to that of the immune exclusive subgroup.

Notably, we found no significant differences between the OS and RFS of patients with high β-catenin/DKK1 and high TIL expression and those with low TIL expression. These results suggest that despite the high CD8 TIL levels in the tumor tissues, the favorable clinical effect of CD8 T cells could be expected from low Wnt/β-catenin signaling activation. Several modulators are associated with T cell infiltration in the TME, and the Wnt/β-catenin signaling molecule is one of the best characterized factors^19,24^. It has been widely accepted that Wnt/β-catenin signaling affects cancer immunosurveillance across various tumor types^19,43^. Tumor-intrinsic Wnt/β-catenin pathway activation impedes antitumor immunity via various mechanisms, including the release of immunosuppressive chemokines, tumor exclusion of dendritic cells, downregulation of innate immune sensors on the tumor cell surface, and suppression of dendritic cell maturation^44^. Interestingly, Wnt/β-catenin signaling is involved in T cell differentiation, effector function, and migration. The differentiation of naïve CD8+ T cells into CD8+ T effector cells is inhibited by the activation of TCF-1/β-catenin signaling^43^, in accordance with the findings of this study. The mechanisms discussed above indicate that the increased expression of β-catenin in tumors is inversely correlated with intratumoral T-cell infiltration. However, we did not find an inverse correlation between tumoral β-catenin or DKK1 expression and the CD8+ TILs. This was similar to the findings of Saleh et al., who reported that Wnt signaling- and β-catenin/TCF complex-associated genes were among the significantly upregulated genes in CD8+ TILs among patients with advanced colorectal cancer^45^. This suggests that the cytotoxic functions of CD8+ effector T cells could be compromised by Wnt/β-catenin signaling. β-catenin or DKK1 protein expression in patients with a high-density of CD8+ TILs in the intra-tumoral area was correlated with poor histological differentiation.

Our data confirmed that the distribution of TILs and the roles of DKK1 and β-catenin differ among GBC, ECC, and ICC. Although several studies have shown similar prognoses among the three BTC subtypes using a traditional merged analysis^2,30,46^, a stratified molecular study found a difference in prognosis among these subtypes. There are several possible explanations for this result. First, the immune escape mechanisms vary according to the type of biliary malignancy. In general, BTC is constantly stimulated by intestinal microbes and toxic materials and possesses an immune tolerance ability that can suppress inappropriate inflammatory responses, in addition to an immune ability that protects against harmful stimuli, such as infection and tumor^19–21^. Therefore, the immunobiology, including the immune escape mechanism, depends on the location of exposure^47^. T-cell receptor clonality, the immune gene signature/RNA repertoire, and major histocompatibility complex class polymorphisms have been extensively investigated in various human cancers, including BTC^47,48^. Goeppert et al. reported that downregulated MHC 1 expression in cancer cells may be associated with a low density of anti-tumor inflammatory cells, such as TILs^30^. This mechanism might underlie the poor prognostic role of DKK1 and β-catenin in GBC and ECC, and the opposite result in ICC.

The present study has some limitations. First, this retrospective analysis was performed with a small sample size, which may be attributed to the complexity of the biological function of the molecules and the different cut-off values used to evaluate the significance of these molecules. However, considering the rarity of BTC, the sample size is adequate to derive significant findings and establish the prognostic impact of the expression of the β-catenin and DKK1 proteins. Second, it is difficult to draw widely applicable conclusions from the results, owing to insufficient numbers of patients in some subgroups classified on the basis of the tumor response patterns. Third, the association between the expression levels of the Wnt/β-catenin gene and DKK1 and distribution of CD8+ TIL were not confirmed by other methods. The amount of archival tissue available for this retrospective study was insufficient to conduct other procedures, such as whole-genome and RNA sequencing. Since the immune signatures and molecular profiling of ICC, ECC, and GBC are different, we focused on identifying the clinical implications at the protein level and conducted analyses using the immunohistochemistry method. To the best of our knowledge, no study has investigated the dynamicity and differential effects of the two molecules (β-catenin and DKK1) in BTC depending on the infiltration of CD8+ TILs. Further research is needed to validate the mechanism and prognostic function of DKK1 and β-catenin according to the heterogeneous immune status in BTC.

## Conclusions

β-catenin or DKK1 protein expression is associated with poor clinical outcomes in patients with resected BTC, whereas high CD8+ TIL levels are associated with good clinical outcomes. Notably, a high CD8+ TIL level with high β-catenin or DKK1 protein expression is associated with an unfavorable prognosis. These findings confirm the differential clinical implications of Wnt/β-catenin proteins according to TIL expression in BTC.

## Supplementary Information

Supplementary Figures.

## Data Availability

The data presented in this study are available on request from the corresponding author. The data are not publicly available due to restrictions e.g., privacy or ethical.
