# Supplementary material for: Prognostic value of Dickkopf-1 and ß-catenin expression according to the antitumor immunity of CD8-positive tumor-infiltrating lymphocytes in biliary tract cancer
Source: Sci Rep. 2022 Feb 4;12:1931. doi: 10.1038/s41598-022-05914-4 (PMC8816896; doi:10.1038/s41598-022-05914-4)
Supplement: Supplementary file 1 — Supplementary Figures. [file 41598_2022_5914_MOESM1_ESM.docx]

Supplementary Material


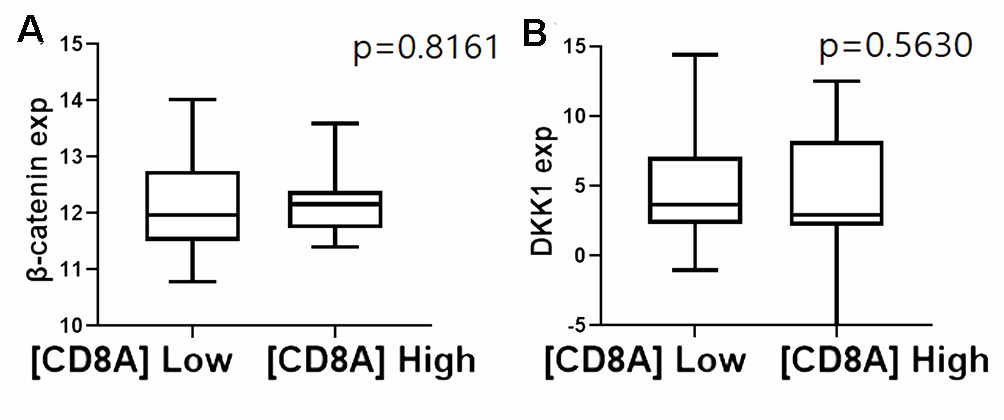


**Supplementary Figure S1.** Correlation among CD8A, DKK1, and CTNNB1 gene expression in BTC samples from TCGA database (n=45).


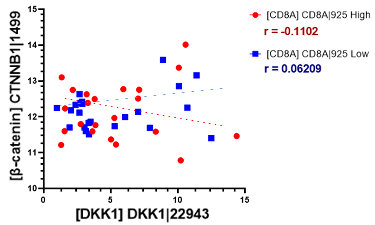


**Supplementary Figure S2.** Correlation among CD8A, DKK1, and CTNNB1 gene expression in BTC samples from TCGA database (n=45).


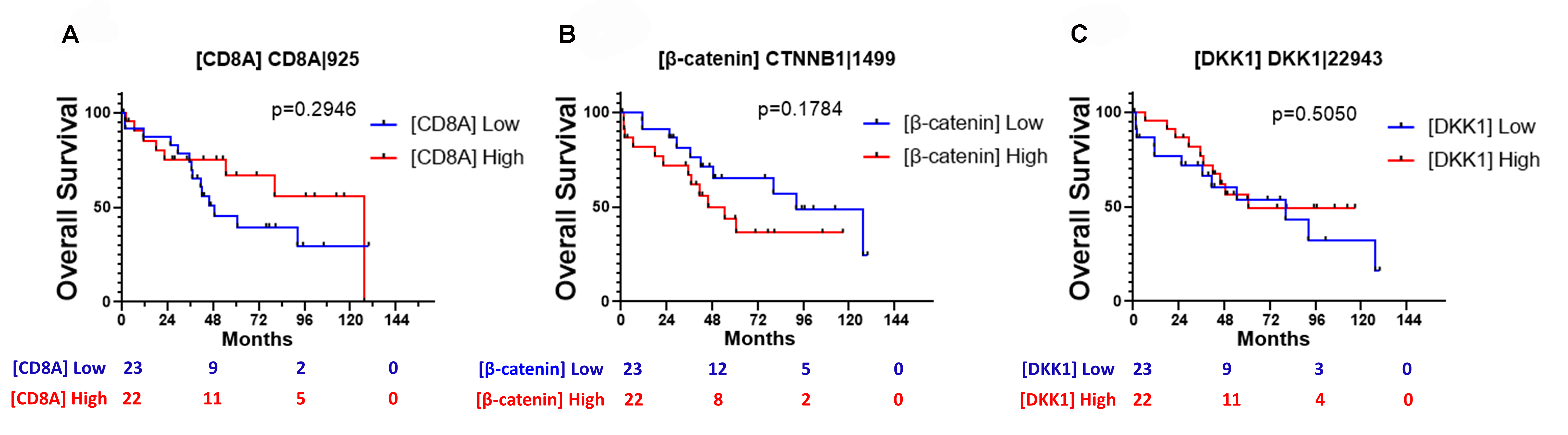


**Supplementary Figure S3.** Survival analysis according to the gene expression of **(A)** CD8A, **(B)** CD8A, and **(C)** DKK1.

**A.** CD8 [CD8A] plot **B.** β-catenin [CTNNB1] plot **C.** DKK1 [DKK1] plot


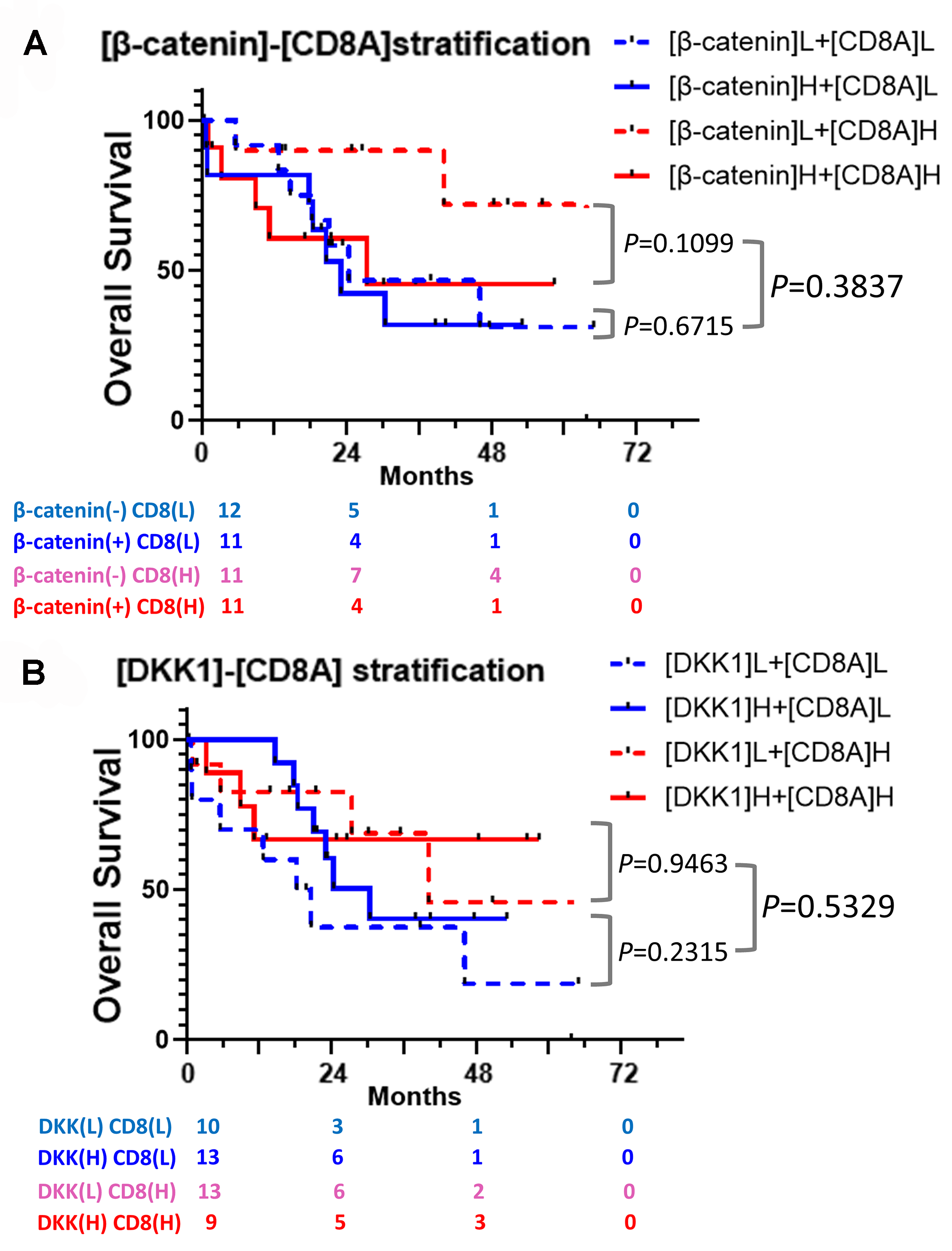


**Supplementary Figure S4.** Survival curves for **(A)** β-catenin and **(B)** DKK1 expression stratified according to CD8A gene expression.

A. β-catenin [CTNNB1] + CD8 [CD8A]

B. DKK1 [DKK1] + CD8 [CD8A]


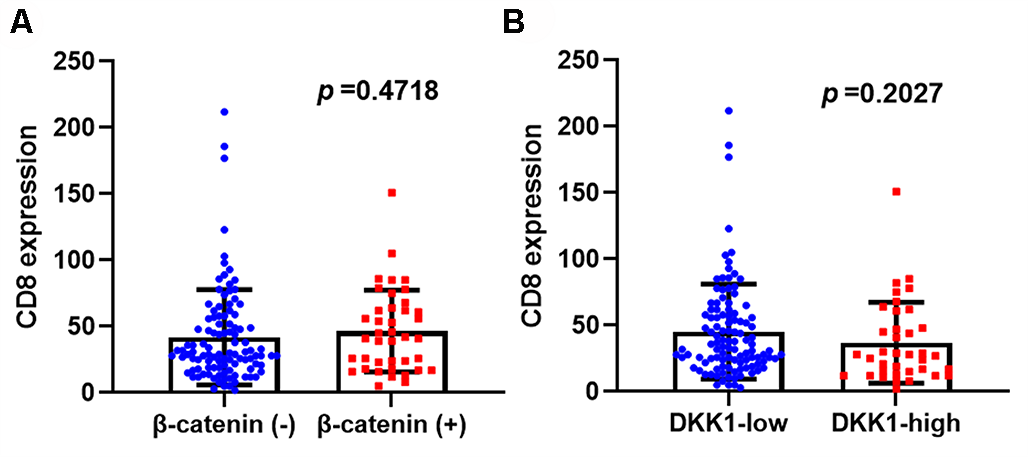


**Supplementary Figure S5.** Correlation between DKK1, ß-catenin, and CD8 expression.

**Supplementary Figure S6.** Overall survival (A, C) and relapse-free survival (B, D) according to ß-catenin, and DKK1 expression.


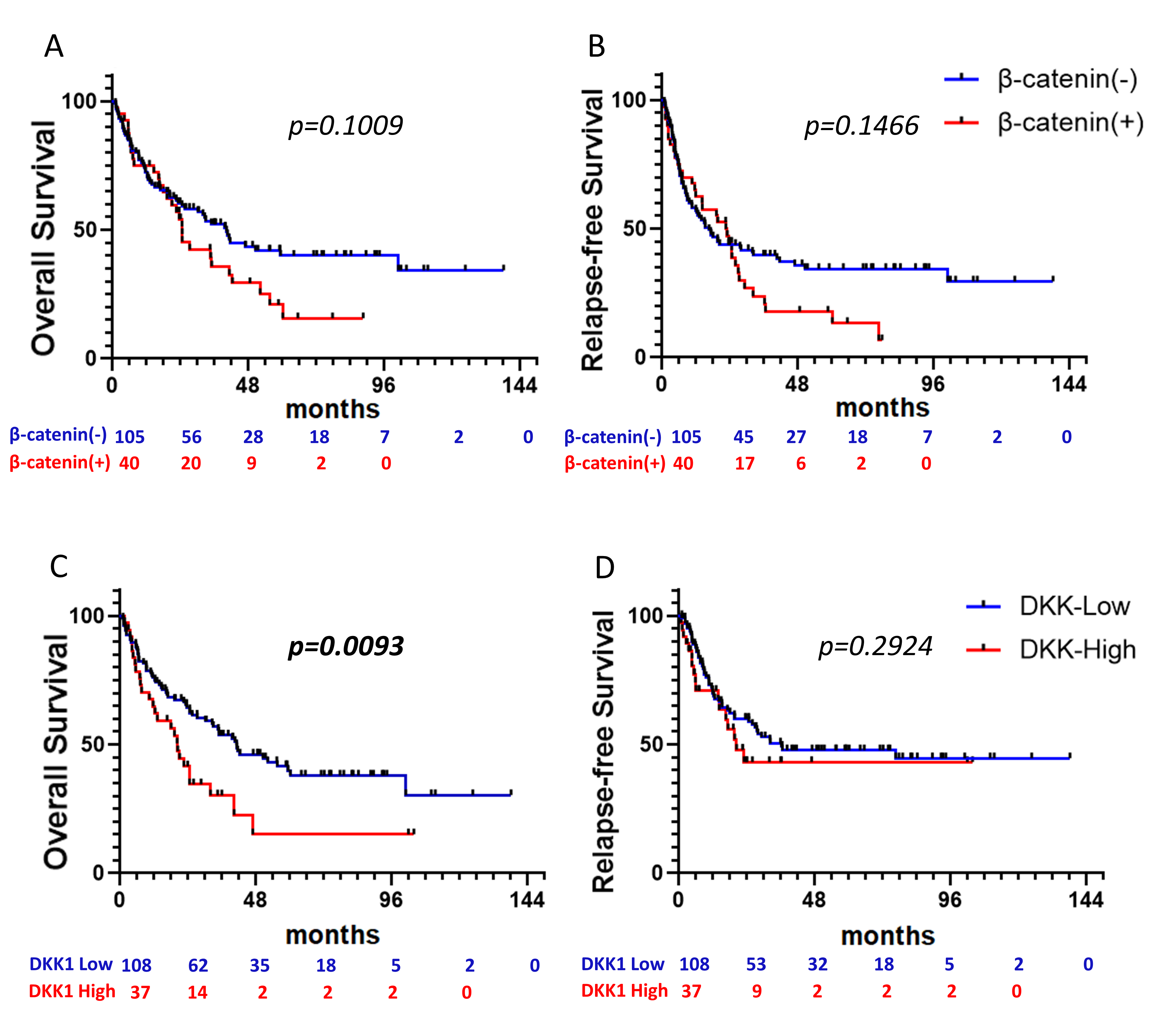


**Supplementary Figure S7**. Comparison of clinical outcomes (overall survival and relapse-free survival) according to the levels of DKK1, beta-catenin, and CD8+TIL expression by primary tumor site.


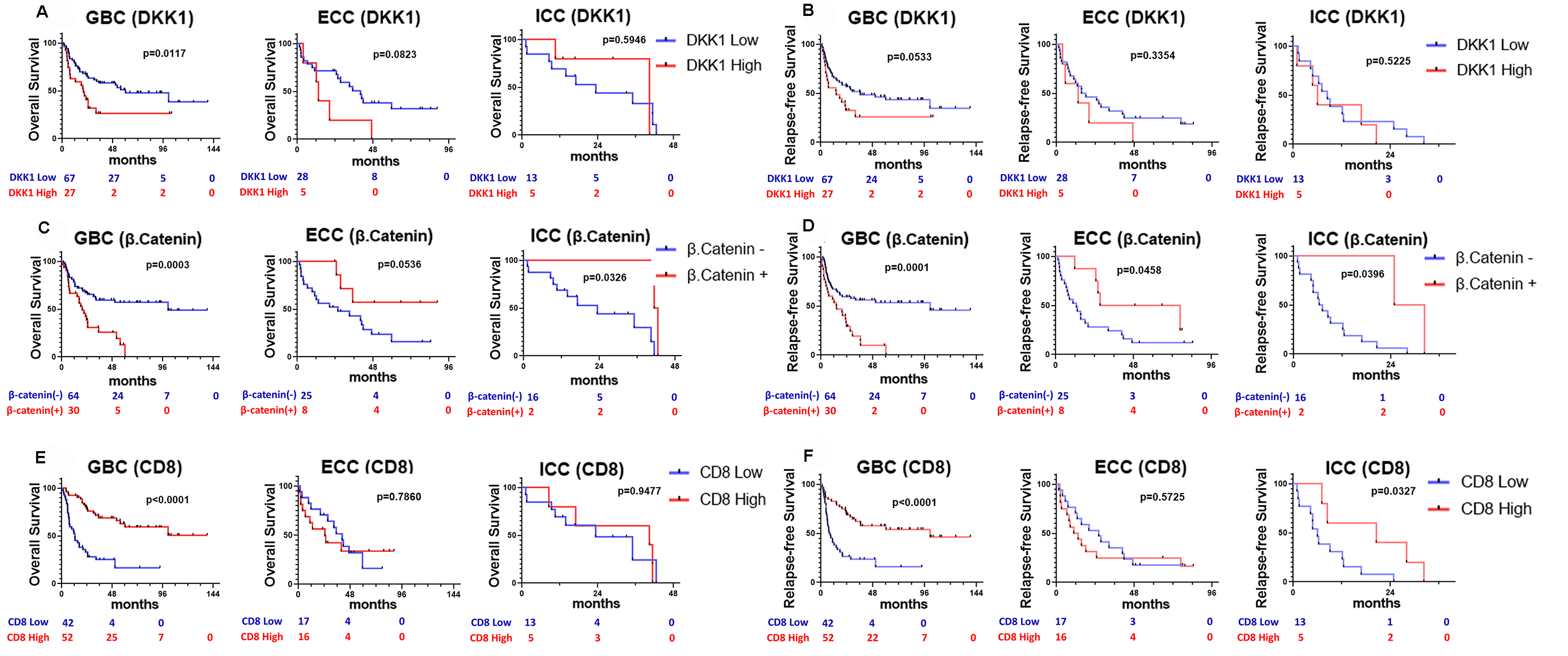


GB, gallbladder; ECC, extrahepatic cholangiocarcinoma; ICC, intrahepatic cholangiocarcinoma

*ECC includes perihilar cholangiocarcinoma, common bile duct cancer, and distal common bile duct cancer.
